# P-1851. OPAT Safety Outcomes with Cefepime vs Piperacillin-Tazobactam

**DOI:** 10.1093/ofid/ofaf695.2020

**Published:** 2026-01-11

**Authors:** Bradley V Dye, Bryan P White, Emily A Siegrist, Maria Alkozah, Joseph Sassine

**Affiliations:** University of Oklahoma, Oklahoma City, OK; OU Health, Oklahoma City, Oklahoma; OU Health, Oklahoma City, Oklahoma; OU Health, Oklahoma City, Oklahoma; University of Oklahoma Health Sciences Center, Oklahoma City, OK

## Abstract

**Background:**

While IDSA guidelines recommend weekly lab monitoring during outpatient parenteral antibiotic therapy (OPAT), cefazolin and ceftriaxone have acceptable safety outcomes with labs assessed every other week. It has not been determined if a similar frequency of lab monitoring could be used with cefepime (FEP) or piperacillin-tazobactam (TZP), nor have these agents been directly compared from a safety standpoint in OPAT.

**Figure:**
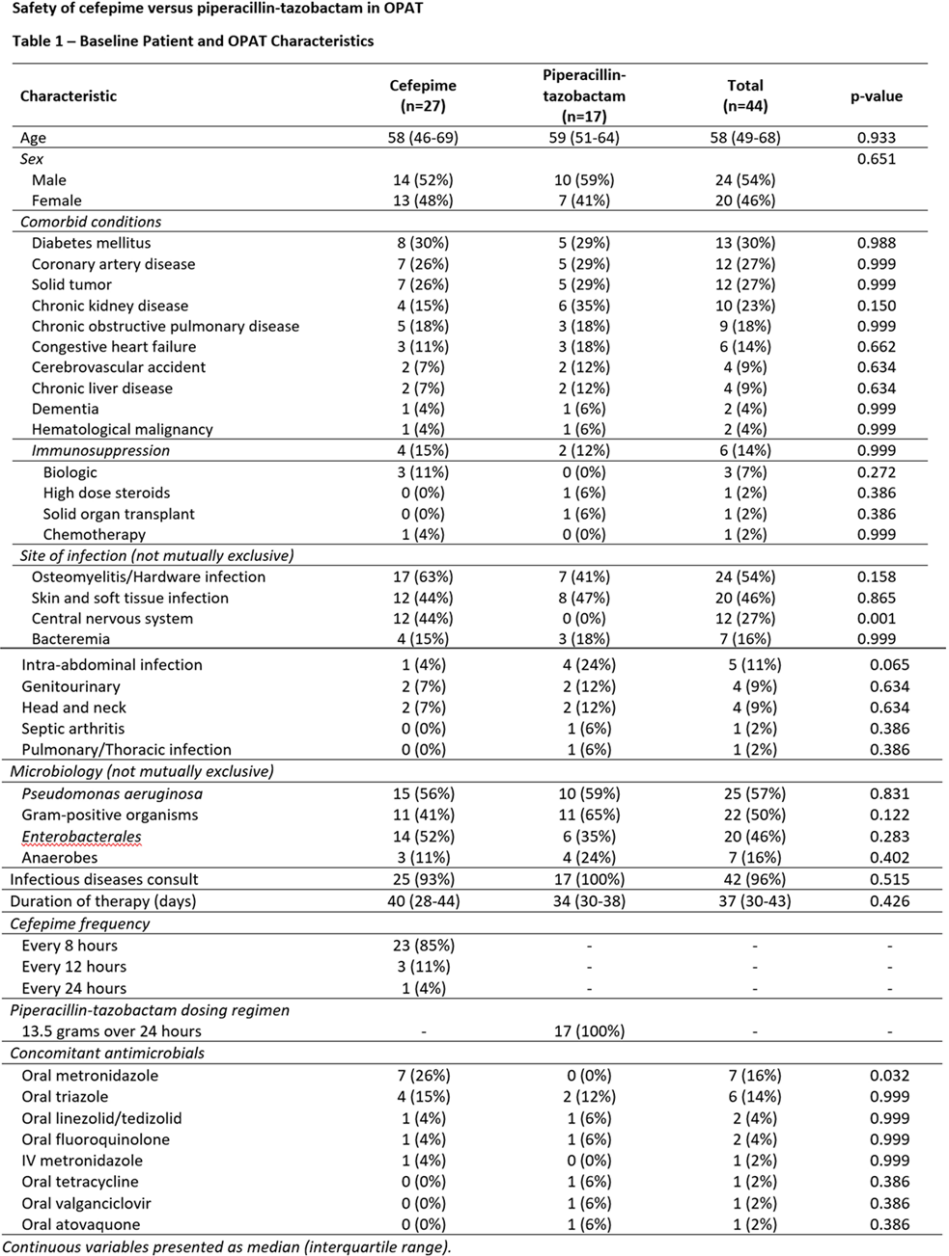

**Figure:**
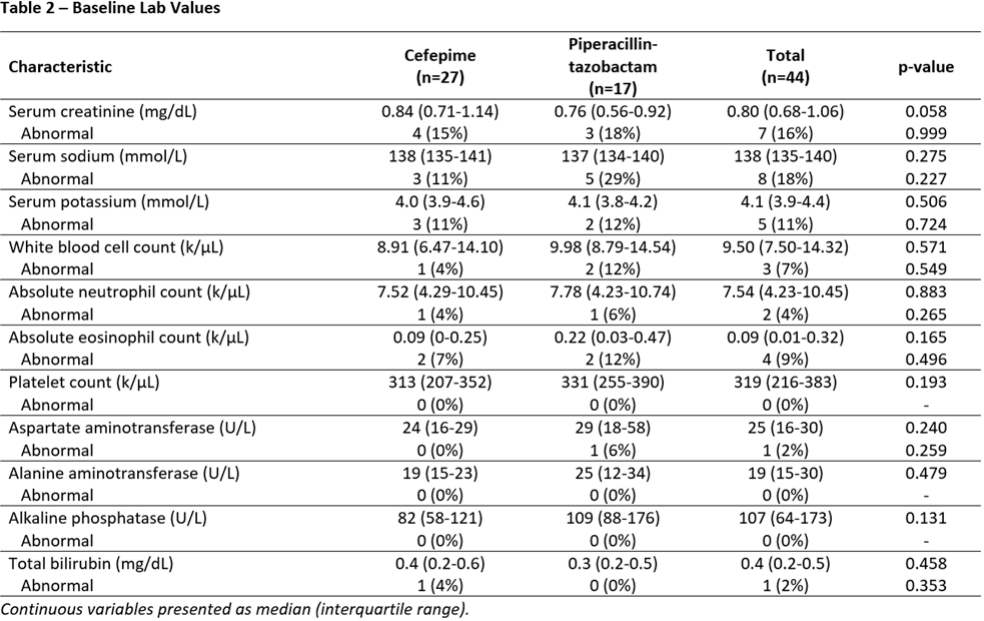

**Methods:**

This is an interim analysis of a retrospective single-center study evaluation of patients receiving FEP or TZP as OPAT between July 2023 and December 2024. Eligible patients were required to complete at least one set of labs during admission and one set of labs during OPAT (complete blood count with basic or comprehensive metabolic panel) with a minimum OPAT duration of 1 week. The primary outcome was the incidence and time to abnormal value for routine lab analytes. Secondary outcomes included change of antibiotic due to adverse events, ER visit or readmission attributed to antibiotic-related adverse events. Statistical analysis was performed via SPSS.

**Figure:**
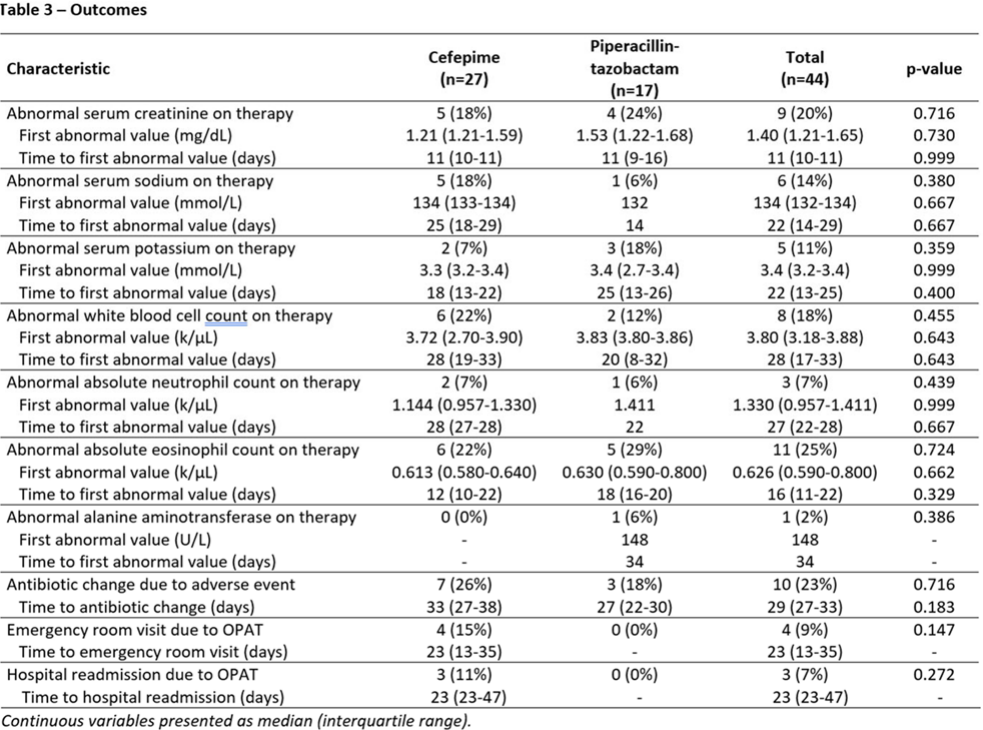

**Figure:**
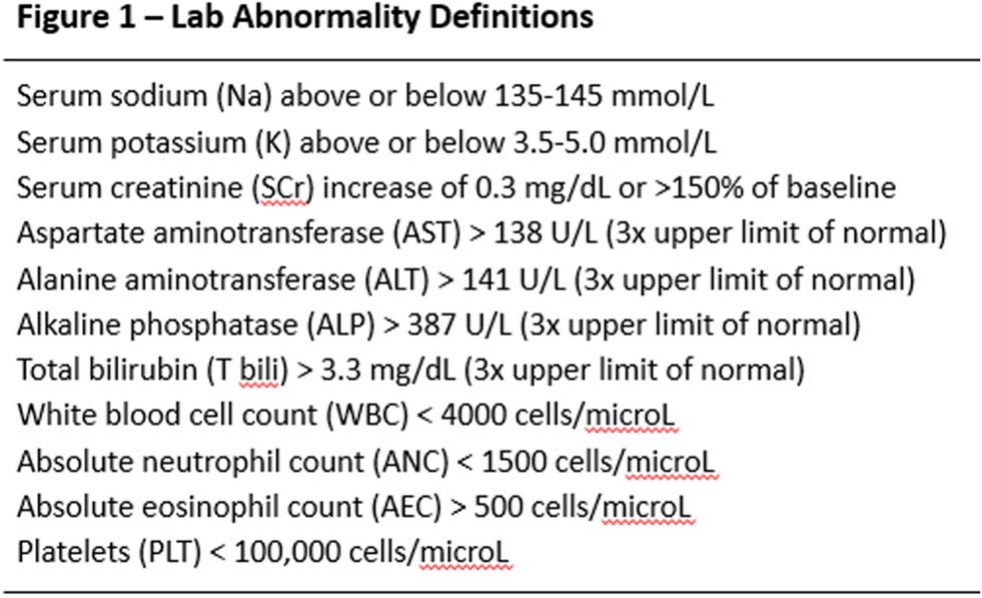

**Results:**

The interim analysis included 44 patients, 27 on FEP and 17 on TZP. There was no difference in baseline labs between the two groups. While no end points comparing FEP and TZP have reached statistical significance, some numerical trends emerged. Leukopenia was more frequent in the FEP group (22% vs. 12%), occurring at a median of 28 days. Increased serum creatinine by > 0.3 mg/dL was more frequent in the TZP group (24% vs. 18%), although the onset was at a median of 11 days for both. The FEP group required more antibiotic changes (26% vs. 18% at median of 33 vs. 27 days) and had more ER visits (15% vs. 0%) and re-admissions (11% vs. 0%) related to OPAT.

**Conclusion:**

Data collection is ongoing to better evaluate safety outcomes between FEP and TZP during OPAT. If distinct risks can be identified with either agent, this may allow better selection of antimicrobials at initiation and lead to fewer safety events during OPAT. Furthermore, if lab abnormalities occur at a predictable time point during therapy, this may allow less frequent checks to reduce the burden on patients and the healthcare system.

**Disclosures:**

Bryan P. White, PharmD, BCIDP, FIDSA, Melinta: Honoraria Joseph Sassine, MD, Ansun Biopharma: Grant/Research Support|Community Infusion Solutions: Grant/Research Support|F2G: Grant/Research Support|Mundipharma: Grant/Research Support|Pulmotect: Grant/Research Support|Shionogi: Grant/Research Support|Viracor: Grant/Research Support

